# The Effect of Liposomes of Various Compositions on the Skin and Its Derivatives After II–IIIA Degree Thermal Burns

**DOI:** 10.32607/actanaturae.27329

**Published:** 2024

**Authors:** N. I. Pashkevich, D. V. Vilyanen, A. F. Marcinkevich, M. M. Borisova-Mubarakshina, S. S. Osochuk

**Affiliations:** Vitebsk State Order of Peoples’ Friendship Medical University, Vitebsk, 210009 Republic of Belarus; Institute of Basic Biological Problems, Russian Academy of Sciences, Pushchino, Moscow Region, 142290 Russian Federation

**Keywords:** burn, skin, phosphatidylcholine, polyunsaturated fatty acid, liposome

## Abstract

This study examines the pathological processes and conditions arising from an experimental modeling of II–IIIA degree thermal burns in laboratory animals. These conditions are characterized by skin structure defects, diminished skin functions, especially the barrier function, and damage to skin derivatives like hair follicles and sebaceous glands. We compared the effect of liposomes composed of soybean lecithin of 90% phosphatidylcholine content and liposomes composed of lecithin of 26% phosphatidylcholine content on the epidermis, dermis and its capillaries, hair follicles, and the sebaceous glands of the laboratory animals 24 h after experimental modeling of II–IIIA degree thermal skin burns. We discuss the dependency of liposome effects on the skin and its derivatives on the fatty acid composition of the lecithin used, with particular focus on phosphatidylinositol, phosphatidic acids, as well as oleic and linoleic acids.

## INTRODUCTION

The skin is known to be the largest organ of the human and animal bodies, and it plays an important role in metabolism. Damage to the skin can cause significant abnormalities in the functioning and condition of all its layers and derivatives, leading to serious changes in the metabolic processes taking place in cells and tissues, or even death.

The human skin contains 3–4 million sweat glands [1]; their total weight is about 100 g [2], which is close to the mean weight of the kidney. Sweat glands are involved in the excretion of xenobiotics, exogenous and endogenous toxic/bioactive substances, such as metals [3], drugs [4, 5], cytokines [6], steroids [7], and lipids, in particular cholesterol [3, 5, 8, 9, 10]. The skin possesses a powerful antioxidant system [2] and is also considered as an independent endocrine organ [11].

Sebaceous glands can act as immunocompetent cells, because they are able to recognize pathogens and synthesize and release pro- and anti-inflammatory cytokines and chemokines, as well as antimicrobial peptides and lipids [12]. An isomer of palmitoleic acid (C16:1D6), sapienic acid in sebum, is known to exhibit antimicrobial activity [13]. Sebaceous gland secretion is a major physiological route of fat-soluble antioxidants to the upper layers of the skin [14]. Additionally, these glands can respond to leptin, linking them to the regulation of starvation and obesity mechanisms [15] and the release of pheromones [16].

Thus, sebaceous glands are considered to be the “brain of the skin” and the most important endocrine glands of the skin. The functional activity of sebaceous glands is closely related to the functioning of hair follicles.

The hair follicle acts as a sensor and immunologic sentinel for the skin. The hair detects stimuli above the skin surface, and the slightest bending of the hair activates neuroreceptors in the follicle, sending sensory information to the nervous system. In turn, Langerhans cells of hair follicles, acting as macrophages, detect surface pathogens and activate the immune system [17]. Hair follicle cells located near the insertion of the erector pili muscle possess the properties of epithelial stem cells and can act as a reserve of epidermal cells and sebaceous glands [18]. Therefore, damage to or preservation of hair follicle cells is an important indicator of the regenerative potential of the skin.

One of the most common skin injuries is burn injury. According to the Federal State Statistics Service of the Russian Federation, the number of burn injury cases in 2021 exceeded 220,000 people [19]. Accordingly, 70% of burn treatments should be performed on an outpatient basis [20]. In this regard, the development of novel drugs for the treatment of burns is of particular topicality.

Healing damaged skin involves four main stages: hemostasis, inflammation, proliferation, and remodeling. Each stage is controlled by a cascade of molecular biological processes [21]. The stage of inflammation and its resolution are considered crucial for wound healing. In this regard, prostaglandins, leukotrienes, and hydroxy- and keto-eicosatetraenoic acids are important [22] and their synthesis requires polyunsaturated fatty acids (PUFAs), mainly omega-3 and omega-6 fatty acids (ω-3 and ω-6, respectively). PUFAs are the preferred targets for free radical oxidation, and their enhanced oxidation can lead to devastating consequences of burn injury [23]. Given the smaller amount of essential PUFAs in phosphatidylcholine from sebaceous glands compared with that in phosphatidylcholine from other organs [24], enhancement of free radical oxidation during skin burns can cause a significant deficiency of essential ω-3 and ω-6 PUFAs. Under these conditions, there may be a partial or significant transition to the production of prostanoids from endogenously synthesized ω-9 series PUFAs capable, under a deficiency of ω-3 and ω-6 series essential fatty acids, of boosting the production of pro-inflammatory cytokines by macrophages [25]. Thus, it seems appropriate to use topical agents containing essential ω-3 and ω-6 PUFAs, such as phosphatidylcholine-based liposomes, in the treatment of burn injuries.

The history of liposome discovery and application began in the 1960s with the work of Alec Bangham and colleagues, who experimented with phospholipids in aqueous media and discovered their ability to form membrane-like structures [26]. Currently, liposomes are widely used as biological nanocontainers for drug delivery in oncology [27, 28], ophthalmology [29], dermatology [30], gene therapy [31], and other fields of medicine. The undeniable advantages of liposomes include their biodegradability, low immunogenicity, and ability to interact with the cell membrane, ensuring intracellular delivery of their contents [32]. However, despite the evidence of high metabolic activity of essential PUFAs constituting the phospholipids used for the production of liposomes, not enough attention is focused on the metabolic effects of such liposomes, depending on the spectrum of fatty acids included in their composition. In the scientific literature, there are only a few studies which compare the activities of liposomes with different fatty acid compositions. For example, L.J. Jenski et al. [33, 34] mentioned the ability of liposomes containing α-linolenic and docosahexaenoic acids to increase the survival chances of mice with experimental cancer. The effect was explained by the cytotoxicity of docosahexaenoic acid against tumor cells. The ability of liposomes composed of linolenic acid-containing phosphatidylcholine to inhibit the growth of *Helicobacter pylori *has been reported [35]. However, there are no systematic studies on the effect of liposomes with different contents and range of fatty acids on skin derivatives.

## EXPERIMENTAL

Our studies were performed on the basis of a cooperation agreement between the Vitebsk State Medical University (VSMU) and the Pushchino Scientific Center for Biological Research of the Russian Academy of Sciences, in the research laboratory of VSMU.

**Preparation of liposomes**

Liposomes of two compositions were prepared from soybean lecithin (phosphatidylcholine) and cholesterol (Sigma, USA) at a 5:1 ratio according to the procedure described in [36]. Lecithin (7.78%) and cholesterol (1.5%) were dissolved in 15 mL of chloroform and 5 mL of methanol. A thin film layer of lipids was formed by vacuum drying the solution on a rotary evaporator. The film was slowly resuspended in 0.01 M phosphate buffer (pH 6) and shaken. The final liposome solution was extruded through a 400 nm filter using an Avanty laboratory mini-extruder (Avanti Polar Lipids, USA). To prepare liposomes of the first composition, we used pharmaceutical lecithin (Riceland Foods, Inc., USA) that contained 26% of phosphatidylcholine, 12–15% of phosphatidylinositol, 4–8% of phosphatidic acids, and 40–50% of free fatty acids; also, 100 g of the compound contained 3,000 mg of P, 1,250 mg of K+, 150 mg of Ca^2+^, 4 mg of Fe, 150 mg of Mg^2+^, 30 mg of Na^+^, and 5 mg of vitamin E. Liposomes of the second composition were prepared using lecithin containing 90% of phosphatidylcholine (PanReac AppliChem, Spain).

**Fig. 1:**
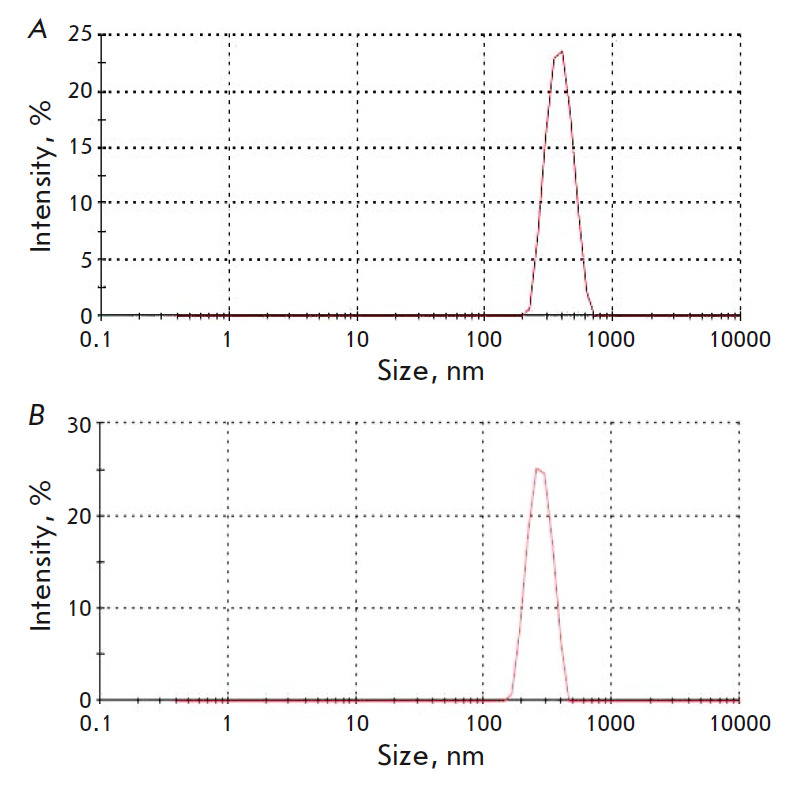
Size distribution of liposomes. (*A*) Liposomes with 26% of phosphatidylcholine. (*B*) Liposomes with 90% of phosphatidylcholine

The size of the liposomes was determined using a Zetasizer Nano ZS analyzer (Malvern Instruments Ltd, UK) (*Fig. 1 A,B*). The mean size of the liposomes containing 26% and 90% of phosphatidylcholine was 486.7 nm and 523.2 nm, respectively.

Fatty acid methylation of both lecithins was performed using sodium methoxide (Sigma-Aldrich). The spectrum of fatty acids was studied by gas-liquid chromatography on a Thermo Focus GC chromatograph (USA) using a capillary column SGEBPX70 (60 m × 0.25 mm) and the temperature program: evaporator temperature – 200°C, flame ionization detector temperature –280°C, the column thermostat temperature was elevated from 120°C to 245°C at a speed of 3°C/min, an isotherm step at 245°C was 5 min (total analysis time was 46.66 min). The carrier gas (He) rate was 1.3 mL/min. Fatty acids were identified with respect to the retention times of standard methyl esters (Sigma-Aldrich). The amount was estimated as a percentage of the total area of all identified peaks.

**Laboratory animals**

The experiments were carried out in two stages, on 92 white non-inbred male rats weighing 180–250 g divided into four groups: 1 – intact animals (*n *= 7 for the first liposome type and *n *= 12 for the second); 2 – stress control, so-called false stress (*n *= 7 for the first liposome type and *n *= 12 for the second) (unburned and untreated animals); 3 – thermal burn (*n *= 15 for the first liposome type and *n *= 12 for the second); and 4 – thermal burn + liposomes of the first type (*n *= 15) and liposomes of the second type (*n *= 12).

**Simulation of thermal burns of the skin**

A device for simulating thermal burns was manufactured by the design bureau Display (Vitebsk, Republic of Belarus) based on a cooperation agreement with VSMU.

**Fig. 2:**
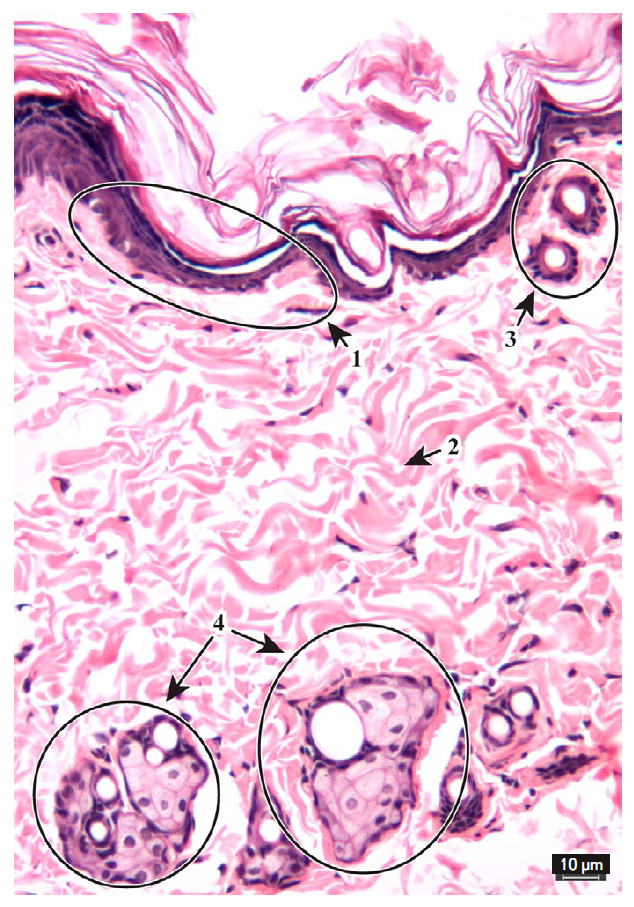
Micrograph of intact animal skin. Hematoxylin and eosin stain. ×20 objective. 1 – epidermis, 2 – dermis, 3 – hair follicles, 4 – sebaceous glands

To simulate burns, the rats were anesthetized with ketamine at a dose of 150 μL/rat [37, 38, 39]. The fur on the back was shaved, and the device heated to 150°C was applied to the skin for 4 min. The induced damage was morphologically assessed as II–IIIA degree burns (*Fig. 2*). The burn area was 8–9% of the body area. To calculate the burn area, we used the formula proposed by Meeh: S = k × W2/3, where S is the body surface area (cm2); W is the body weight of the animal (kg); and k is a Meeh constant of 9.46 [40]. Immediately after burn induction, the damaged skin in the animals of the fourth experimental group was treated with 0.45 mL of a type 1 or type 2 liposome solution.

**Histological examination of the skin**

The animals were decapitated under ether anesthesia 24 h after the burn injury, and the skin was sampled for histological examination. Skin samples were fixed in a 10% neutral formaldehyde solution. Histological sections were stained with hematoxylin–eosin and examined on a Leica DM 2500 microscope (Germany, ×10 eyepiece, ×20 and ×40 objectives) equipped with a Leica DFC 320 digital camera. The number of damaged elements was counted in 10 fields of view, and the mean indicator of examined skin elements was calculated.

**Statistical data analysis **was performed using the R package, version 4.0.5 (2021-03-31). The distribution of analyzed indicators was assessed using the Shapiro–Wilk test; in the case of a Gaussian distribution, parametric statistics methods were used for comparison; otherwise, nonparametric methods were used. Pairwise comparisons were performed using the Student’s *t *test or the Wilcoxon–Mann–Whitney test. Multiple comparisons were performed using ANOVA (in the case of heterogeneity of variances of the analyzed indicators, the Welch correction was applied) or the Kruskal–Wallis H test. *Post hoc *analysis was performed using the Tukey test or the Kruskal–Wallis H test and the Dunn test, corrected for multiple comparisons using the Benjamini–Iekutieli method. Differences were considered statistically significant at *p* < 0.05.

## RESULTS

According to our measurements using gas-liquid chromatography, pharmaceutical lecithin (26% phosphatidylcholine) included the following fatty acids (of the total fatty acid content, %): C16:0 – 18.87, C18:0 – 3.86, C18:1n9c – 8.8, C18:2n6c – 56.95, and C18:3n3 – 6.81. Lecithin containing 90% of phosphatidylcholine included the following fatty acids (of the total fatty acid content, %): C16:0 – 13.63, C18:0 – 3.68, C18:1n9c – 11.36, C18:2n6c – 62.88, and C18:3n3 – 6.12. Therefore, lecithin containing 90% of phosphatidylcholine had a higher content of linoleic acid (C18:2n6c – 62.88% vs. 56.95% in pharmaceutical lecithin) and oleic acid (C18:1n9c 11.36% vs. 8.8% in pharmaceutical lecithin).

An assessment of the influence of the so-called false stress (see Experimental section) on the examined indicators did not reveal statistically significant deviations from the values characteristic of the intact animals (*Table 1*).

**Table 1:** Effect of liposomes with various compositions on the parameters of the skin and its derivative 24 h after burn injury

Intact	False stress	Burn	Burn + liposomes with 26% PC	Burn + liposomes with 90% PC
Epidermis thickness, μm				
28.89 ± 3.63	25.95 ± 5.97	13.00 ± 3.87^*,#^	11.93 ± 2.69^*,#^	12.25 ± 4.03^*,#^
Thickness of the stratum corneum of the epidermis, μm				
13.05 ± 2.04	13.42 ± 3.91	5.70 ± 2.20^*^	4.73 ± 1.49^*,#^	5.00 ± 2.95^*,#^
Epidermis thickness (without stratum corneum), μm				
15.84 ± 2.73	12.53 ± 2.46	7.30 ± 2.32^*^	7.20 ± 2.14^*,#^	7.25 ± 1.71^*,#^
Number of hyperemic vessels				
0.00 ± 0.00	0.00 ± 0.00	14.48 ± 4.73^*,#^	12.00 ± 3.74^*,#^	7.75 ± 1.22 ^*,#,v^
Number of damaged hair follicles				
0.00 ± 0.00	0.00 ± 0.00	2.93 ± 3.47^*,#^	0.00 ± 0.00^v^	3.42 ± 0.90^*,#,a^
Number of damaged sebaceous glands				
0.00 ± 0.00	0.00 ± 0.00	6.78 ± 1.19^*,#^	6.13 ± 1.13^*,#^	0.00 ± 0.00^v,a^

Note. PC – phosphatidylcholine. Statistically significant: * – compared with intact rats; # – compared with stress; v – compared with burn, a – compared with 26% PC.

The epidermis of the intact animals and animals from the false stress group has a layered structure: the stratum corneum, basal, spinous, and granular layers are preserved (*Fig. 2*). The dermis is characterized by a fibrous structure; collagen fibers are crimped; the tissue is well structured. The integrity of hair and hair follicles is preserved; the outer and inner epidermal root sheath is clearly structured; and the medulla is not expanded. The integrity of sebaceous glands is preserved; secretion-containing vesicles are tightly adjacent to each other; and the stratified epithelium of the glands is preserved. Microvasculature vessels and papillary capillaries of the dermis are not hyperemic and, therefore, are poorly visualized.

**Fig. 3:**
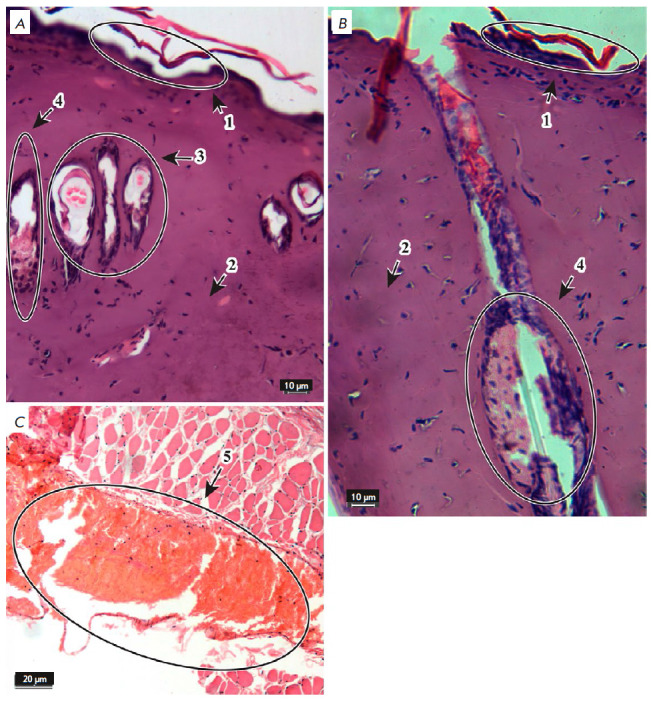
Morphological changes in the skin 24 h after thermal burn. Hematoxylin and eosin stain. ×20 (*A*, *B*) and ×40 (*C*) objectives. 1 – epidermis, 2 – dermis, 3 – hair follicles, 4 – sebaceous glands, 5 – blood vessels

*Figure 3* shows the changes in the skin 24 h after the II–IIIA degree thermal burns. The structure of all epidermal layers had changed, which manifested itself as the destruction of the stratum corneum and necrosis of highly proliferating cells of the spinous and basal layers; the granular layer was preserved only in certain areas; the epidermal–dermal junction was flattened. There were no dermal papillae or dermal fiber bundle structure; the tissue was represented by a homogeneous structureless mass resulting from collagen denaturation. Destruction of the hair root, root sheath, and connective tissue follicle was not observed; the medulla of the hair root was significantly expanded, the shape of the hair root was changed, and there was a single-layer cell contour around the root. Sebaceous glands were deformed, secretory vesicles were destroyed, and epithelial cells were damaged. Hypodermal vessels were hyperemic, and the wall was destroyed.

A mathematical assessment of the burn impact on the assessed parameters revealed a statistically significant decrease in the thickness of the epidermis, stratum corneum of the epidermis, and epidermis without the stratum corneum compared with that in both intact and false stress animals (*Table 1*). In addition, the number of hyperemic vessels, hair follicles, and sebaceous glands was increased in the group of animals with burns compared with that in intact and false stress animals; accordingly, burns caused negative, statistically significant changes in all analyzed parameters (*Table 1*).

**Fig. 4:**
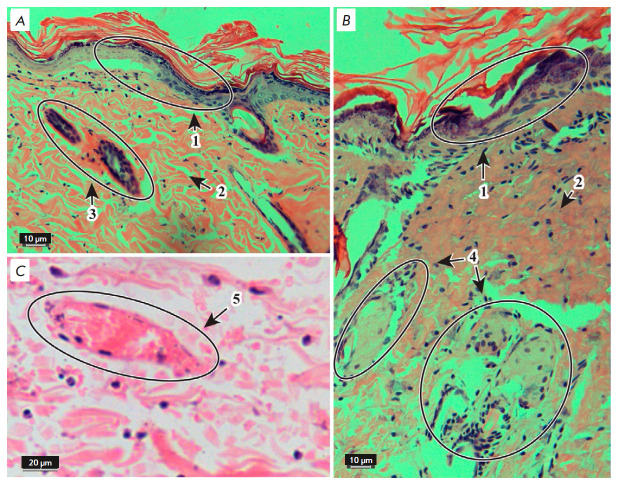
Morphological changes in the skin with burn injury 24 h after application of liposomes with 26% of phosphatidylcholine. Hematoxylin and eosin stain. ×20 (*A*, *B*) and ×40 (*C*) objectives. 1 – epidermis, 2 – dermis, 3 – hair follicles, 4 – sebaceous glands, 5 – blood vessels

the epidermis was noted 24 h after burning and application of a solution of lecithin liposomes containing 26% of phosphatidylcholine to the burned surface (*Fig. 4*). There was partial formation of dermal bundles, a crimped fiber structure, an increased number of microvessels, and, compared with the skin of the intact animals, a large amount of amorphous substance. The integrity of the hair and hair follicles was preserved, the epidermal root sheath was structured, and the medulla was not expanded. The secretory vesicles of sebaceous glands were damaged, the epithelial cells were loosely located, and intercellular contacts were lost. Large hypodermal vessels were hyperemic, and their walls were partially destroyed.

**Fig. 5:**
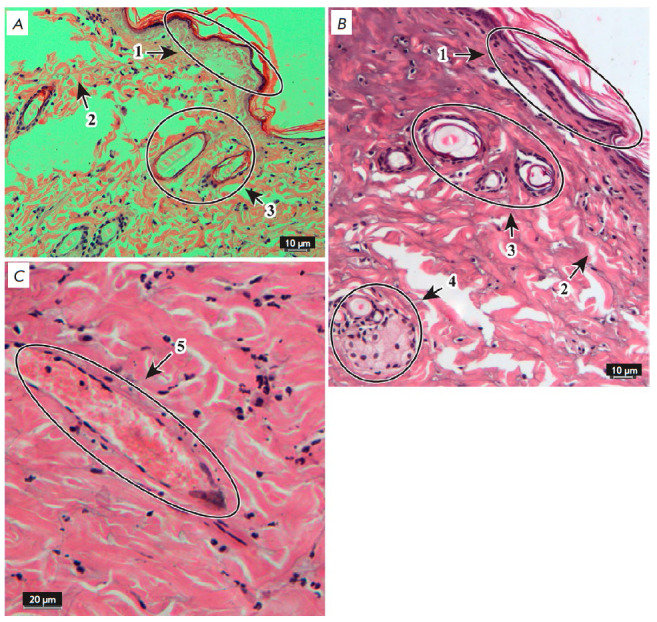
Morphological changes in the skin with burn injury after application of liposomes with 90% of phosphatidylcholine. Hematoxylin and eosin stain. ×20 (*A*, *B*) and ×40 (*C*) objectives. 1 – epidermis, 2 – dermis, 3 – hair follicles, 4 – sebaceous glands, 5 – blood vessels

Following application of a solution of lecithin liposomes containing 90% of phosphatidylcholine, a partial restoration of the layered epidermal structure, an increased number of basal layer cells, a decreased number of spinous layer cells, and a damaged granular layer were observed (*Fig. 5*). There was partial formation of dermal bundles, a crimped fiber structure, an increased number of microvessels, and, compared with the skin of the intact animals, a large amount of amorphous substance. The hair root and outer and inner root sheath were destructed, the medulla of the hair root was expanded, the shape of the hair root was changed, and there was a single-layer contour of cells around the root. The integrity of sebaceous glands was preserved, the secretion-containing vesicles were tightly adjacent to each other, and the stratified epithelium of the glands was preserved. Large hypodermal vessels were hyperemic, and their walls had partially thinned.

According to a mathematical assessment, the liposomes of different fatty acid compositions did not have a statistically significant effect on the thickness of the epidermis, stratum corneum of the epidermis, or epidermis without the stratum corneum, but there was a trend towards a partial restoration of the epidermal layers upon using lecithin containing both 26% and 90% of phosphatidylcholine (*Table 1*). However, no damaged hair follicles were detected after the application of liposomes composed of lecithin of 26% phosphatidylcholine content (*Table 1*).

Liposomes composed of lecithin containing 90% of phosphatidylcholine had no effect on the number of damaged hair follicles, but it reduced the number of hyperemic vessels by 47% compared with that in the group of burned animals and eliminated the negative effect of burns on sebaceous glands, whose number was reduced to values observed in healthy animals (*Table 1*).

Therefore, the burns caused negative changes in all studied parameters. Liposomes composed of lecithin containing 26% of phosphatidylcholine prevented the development of damage to hair follicles, which may be due to additional components present in pharmaceutical lecithin.

Liposomes composed of lecithin containing 90% of phosphatidylcholine reduced the number of hyperemic blood vessels and prevented damage to sebaceous glands. In addition, a significant restoration of the dermis structure was observed with liposomes composed of lecithin containing both 26% and 90% of phosphatidylcholine.

## DISCUSSION

The barrier function is known to be the main role of the skin. This function is actualized by preventing the physical penetration of foreign components by keratinocytes, due to the unity of their monolayers; the lipid- protective part due to the presence of ceramides, cholesterol, and the free fatty acids present in the intercellular space of corneocytes; lipolytic, proteolytic enzymes, and antimicrobial peptides synthesized by skin cells; and the renewal of lipid and enzymatic components through their secretion by skin cells [41, 42].

Our findings suggest that burns significantly reduce the barrier function of the skin, in particular due to the lipid-protective component of the intercellular space of corneocytes. Probably, inclusion of lipid components into anti-burn agents may reduce the intensity of thermal damage to the skin. Also, phosphatidylinositol 3-kinase/protein kinase B is known to be involved in postburn sepsis [43]. The presence of 12–15% of phosphatidylinositol (phosphatidylinositol 3-kinase/protein kinase B substrate) in 26% of lecithin suggests potentiation of the negative effect of burn injury. However, our experiment revealed no negative effects from the first type of liposomes on the studied parameters, suggesting that phosphatidylinositol does not adversely affect the burn healing process. The positive effect of liposomes composed of 26% of phosphatidylcholine on preventing damage to hair follicles may be due to the phosphatidic acids present in the substance from which the liposomes are made (4–8%). In this regard, the lack of a positive effect from liposomes composed of lecithin with 90% of phosphatidylcholine on hair follicles may be due to the absence of phosphatidic acids in the liposomes. This suggestion requires further research.

The observed decrease in the number of hyperemic vessels is regarded as positive, because the intensity of a skin vessel filling up with blood cells is associated with inflammatory process activity [44]. Thus, liposomes composed of lecithin containing 90% of phosphatidylcholine likely have the ability to reduce inflammatory activity. This effect may be related to the higher content of γ-linolenic acid (C18:3n6c) in lecithin containing 90% of phosphatidylcholine, which is consistent with its positive effects reported in experimental burns in rats [45].

Sebaceous glands are known to release their secretion into hair follicles [46, 47] and synthesize a mixture of lipids, creating a permeability barrier and imparting certain antimicrobial properties to the skin [48, 49]. For this reason, preservation of sebaceous glands by liposomes composed of lecithin containing 90% of phosphatidylcholine is a considerable protective outcome. This effect is most likely due to a higher phosphatidylcholine content and higher contents of linoleic acid (C18:2n6c, 62.88% vs. 56.95%) and oleic acid (C18:1n9c, 11.36% vs. 8.8%) than in pharmaceutical lecithin. This suggestion seems reasonable, because there is evidence that oleic (C18:1n9) and linoleic (C18:2n6) acids are able to accelerate the healing of skin wounds and reduce the intensity of any inflammation in them by inhibiting the production of pro-inflammatory cytokines [50, 51]. Regeneration of auxiliary organs of the skin, such as hair follicles and sebaceous glands, not only accelerates wound healing, but also improves the functionality of regenerated skin. Furthermore, regeneration of sebaceous glands indirectly indicates a regeneration of the hair follicle [52].

## CONCLUSION

The presented material suggests that liposomes composed of lecithin containing 90% of phosphatidylcholine are more effective in reducing vascular hyperemia in burn-damaged skin and in preventing damage to sebaceous glands than those composed of lecithin containing 26% of phosphatidylcholine. Nevertheless, liposomes composed of lecithin with 26% of phosphatidylcholine prevent damage to hair follicles, which may be due to the presence of phosphatidic acids, as well as other components. Further research is needed to elucidate the molecular mechanisms of action of liposomes of various compositions.

## Abbreviations

PUFA: polyunsaturated fatty acid
